# Structural Analysis of Human LonP1 Protease Bound with the Native Substrate

**DOI:** 10.3390/life16030478

**Published:** 2026-03-16

**Authors:** Ming Li, Hongwei Liu, Shengchun Zhang, Qijun Gao, Shanshan Li, Junfeng Wang, Kaiming Zhang

**Affiliations:** 1 Division of Life Sciences and Medicine, University of Science and Technology of China, Hefei 230026, China; bright233@ustc.edu.cn (M.L.); z1140729264@163.com (H.L.); chunchunzhang@mail.ustc.edu.cn (S.Z.); gqj@mail.ustc.edu.cn (Q.G.); lishanshan@ustc.edu.cn (S.L.); 2 High Magnetic Field Laboratory, Hefei Institutes of Physical Science, Chinese Academy of Sciences, Hefei 230031, China; 3 Key Laboratory of High Magnetic Field and Ion Beam Physical Biology, Hefei Institutes of Physical Science, Chinese Academy of Sciences, Hefei 230031, China

**Keywords:** Lon protease, cryo-EM, native substrate

## Abstract

The human mitochondrial Lon protease (LonP1) is a central regulator of mitochondrial DNA copy number and metabolic reprogramming. However, the structural basis for how LonP1 recognizes native physiological substrates remains elusive. Here, we present the high-resolution cryo-EM structure of the human LonP1 hexamer actively engaging its native substrate, TFAM. The reconstruction reveals a distinct bipartite search-and-shred mechanism. Unlike its bacterial homologs, the human N-terminal domain (NTD) adopts a compact architecture acting as a selective vestibule to recruit and initially unfold the substrate tertiary structure. Subsequently, the polypeptide is threaded through the central channel via a hand-over-hand mechanism driven by a spiral array of aromatic pore-loops. This structural framework provides a mechanistic rationale for the spatial segregation of LonP1 and offers a template for targeting mitochondrial proteostasis in human diseases.

## 1. Introduction

Mitochondrial homeostasis depends on a stringent Protein Quality Control (PQC) network that counteracts proteotoxic stress and maintains the fidelity of oxidative phosp. horylation (OXPHOS) [1,2]. At the center of this network is the homo-hexameric human Lon protease (LonP1), an ATP-dependent AAA+ protease. LonP1 functions not only as a degradative machinery for oxidized proteins [3], but also as a key regulatory hub that governs the mitochondrial DNA copy number and metabolic reprogramming by selectively turning over specific native substrates, including the mitochondrial transcription factor A (TFAM) [4] and multiple metabolic enzymes [5,6]. Consequently, LonP1 dysfunction is inextricably linked to severe pathologies, including CODAS syndrome [7,8], neurodegenerative disorders [9,10], and cancer [11], making it a pivotal target for therapeutic intervention [12,13].

Given its clinical importance, substantial efforts have been devoted to elucidating the molecular architecture of LonP1. Pioneering studies utilizing X-crystallography to resolve the proteolytic domain [14] and subsequent cryo-electron microscopy (Cryo-EM) studies resolved structures of human LonP1 in its apo state, ADP-bound forms, or in complex with synthetic peptides and proteasome inhibitors (Bortezomib) [3,15]. Together, these pioneering works have established a foundational understanding of the spiral arrangement of the ATPase domains [16] and the hand-over-hand translocation mechanism [17]. They have eloquently described how LonP1 mechanically unfolds and threads polypeptides through its central channel for degradation [18].

However, a critical gap persists in bridging structural mechanics with physiological specificity. The majority of available structures rely on short synthetic degrons or non-native model substrates like casein to mimic substrate engagement [15,19]. Although these systems have been invaluable for dissecting translocation mechanics, they do not recapitulate the initial and specific recognition and conformational changes that occur between LonP1 and its globular native partners. In particular, the N-terminal domain (NTD) of human LonP1 exhibits significant sequence and structural divergence from its bacterial homologs. Unlike the well-characterized N-domain of bacterial LonA, which forms a stable bilobed structure for substrate interaction [18,20], the human NTD is largely unique and structurally elusive. Whether this divergent NTD adopts a specific conformation to distinguish a specific physiological target from the broader mitochondrial proteome [21], and how the interaction with a native, full-length substrate allosterically activates the protease prior to translocation, remains largely obscure.

This absence of structural information at the native recognition interface represents a major bottleneck for structure-guided drug design [22], constraining the development of highly selective inhibitors to reduce the potential for off-target toxicity [23,24]. To address these limitations, we determined a high-resolution cryo-EM structure of human LonP1 in complex with its authentic native substrate, TFAM. Our structures elucidate how the native substrate undergoes unfolding within the central channel of LonP1 and engages in extensive interactions spanning from the N-terminal region to the AAA+ domain, and the N-terminal region of LonP1 displays remarkable conformational flexibility and structural distinctness from bacterial homologs. This study provides the atomistic insights into how human LonP1 engages and unfolds a physiological substrate and offers a novel template for designing next-generation allosteric inhibitors [25] or PROTACs [26] that can precisely modulate LonP1 activity in disease contexts. Thus, this work not only advances our fundamental understanding of mitochondrial PQC but also lays the structural foundation to overcome current barriers in targeting mitochondrial proteases [11,27] for clinical applications.

## 2. Materials and Methods

### 2.1. Cloning and Purification of Human LonP1

The gene of human LonP1 protease (human LonP1, NCBI accession: NM_004793.4) without mitochondria targeting sequence (MTS:1-114) was codon-optimized and cloned into a pET28a vector, followed by a 6× His tag at the C-terminus (human LonP1-His). The human LonP1 mutant (T564A-Y565A-V566A) was constructed by overlap PCR with the primers (Mutant F: CACAGGCGGgcggcagcaGGCGCCATGCCCGGGAAG, Mutant R: tgctgccgcCCGCCGCCTGTGGCCCTTGATCTCAG). The plasmid pET28a-human LonP1-His and pET28a-human Lonp1 mutant were generated in *E*. *coli* Top10 competent cells and transformed into *E*. *coli* BL21(DE3) pLysS competent cells to produce protein. The cells were then grown in Luria–Bertani media at 37 °C until the OD600 reached 0.8. After that, 0.5 mM IPTG was added to the media, and the cells were cultured for another 16–20 h at 16 °C. The cells were harvested via centrifugation (6000 rpm,10 min) and lysed by sonication in lysis buffer containing 50 mM Tris (pH 7.8), 500 mM NaCl, and 5% glycerol. The supernatant of the lysate was incubated with 5 mL nickel resin (Cytiva) for 0.5 h before applying to a column. The resin was washed with 40 mL lysis buffer, 40 mL of lysis buffer supplemented with 20 mM imidazole, and 40 mL of lysis buffer supplemented with 40 mM imidazole. The protein was eluted by lysis buffer supplemented with 400 mM imidazole. The elution was pooled and concentrated by 100 kDa cut-off and loaded into a Superose6 increase 10/300 increase GL column (Cytiva) pre-equilibrated with gel filtration buffer (25 mM Tris (pH 7.5), 150 mM NaCl, 1 mM DTT). The peak fractions were collected, pooled, and flash frozen for further use.

The gene of transcription factor A (TFAM, Uniprot accession code: Q00059) was codon-optimized and cloned into a pET22b vector with a 6× His tag at the N-terminus (His-TFAM). The plasmid pET22b-His-TFAM was generated in *E. coli* Top10 competent cells and transformed into *E*. *coli* BL21(DE3) pLysS competent cells to produce protein. The cells were then grown in Luria–Bertani media at 37 °C until the OD600 reached 0.8. After that, 0.5 mM IPTG was added to the media, and the cells were cultured for another 16–20 h at 16 °C. The cells were harvested via centrifugation (6000 rpm, 10 min) and lysed by sonification in lysis buffer containing 20 mM HEPES (pH 8.0), 500 mM KCl, and 5% glycerol. The supernatant was collected after centrifugation (12,000 rpm, 1 h) and incubated with 5 mL nickel resin (Cytiva) for 0.5 h before applying to a column. The resin was washed with 50 mL lysis buffer, 50 mL of lysis buffer supplemented with 20 mM imidazole, and the protein was eluted with lysis buffer supplemented with 400 mM imidazole. The protein was concentrated by a 10 kDa cut-off and loaded into a Superdex75 increase 10/300 GL column. The peaked fractions were collected, pooled and flash frozen for further use.

### 2.2. Negatively Stained TEM and Cryo-EM Sample Preparation

The protein was diluted to 0.03 mg/mL with gel filtration buffer and centrifuged at 13,000 rpm for 10 min, and three microliters of supernatant were applied on the glow-discharged grids by PELCO easiGlow for 30 s, and removed the excessive sample by a filter paper and stained with 2% uranyl acetate for 1 min. The excessive uranyl acetate was removed by a filter paper and dry on the room temperature. The grids were loaded into a 200 kV Tecnai TF20(FEI) electronic microscope (Tecnai TF20, FEI, Hillsboro, OR, USA).

Before preparing cryo-EM grids, the human LonP1-His (3 μM) was incubated with 15 μM TFAM supplemented with 10 mM MgCl_2_, 5 mM ATP*γ*S and 1 mM Bortezomib on ice for 0.5 h. Three microliters of supernatant of the mixture were applied on the glow-discharged Quantifoil R2/1 200-mesh copper grids (Quantifoil, Micro Tools GmbH, Braunschweig, Germany). After 5 s incubation, grids were blotted for 3.5 s (blot force 0) at 4 °C and 100% humidity, then plunge-frozen in liquid ethane using a Vitrobot Mark IV (Thermo Fisher Scientific, Waltham, MA, USA). Grids were stored in liquid nitrogen before screening on an electron microscope.

### 2.3. Cryo-EM Data Collection and Image Processing

The grids were initially screened using a Glacios microscope (Thermo Fisher Scientific) operated at 200 kV, followed by high-resolution data collection on a Titan Krios G3i (Thermo Fisher Scientific) at 300 kV, equipped with a K3 Summit direct electron detector (Gatan, Pleasanton, CA, USA). Images were acquired using EPU software (version 2.7) at a magnification of 105,000×, corresponding to a calibrated pixel size of 0.82 Å (Table 1). Each exposure lasted 1.7 s with an electron dose rate of 26.46 e^−^/Å^2^/s.

For cryo-EM data processing, a standard cryoSPARC pipeline [28] was employed. A total of 4890 dose-fractionated movies underwent patch-based motion correction and contrast transfer function (CTF) estimation, followed by particle extraction using a box size of 384 pixels. After 2D classification, ab initio 3D reconstruction, and heterogeneous refinement, 99,919 particles (belonging to class 1 of 3) were selected for non-uniform refinement and local refinement, yielding a final whole map at 3.22 Å resolution and a local map (NTD) at 4.56 Å (Figure S2).

### 2.4. Model Building and Refinement

The sequence of human LonP1-His was imported into the AlphaFold3 server [29] for the initial model generation of their protomers, respectively. The initial models of protomers were first rigidly fitted into the cryo-EM maps using ChimeraX-1,10 [30], applying “fit in map tool”, and molecular dynamics flexible fitting (MDFF) was applied to flexibly fit the atomic model into the map, followed by optimization with Coot [31] and phenix.real_space_refine. The substrate was manually adjusted by Coot and refined by phenix.real_space_refine. The final models were evaluated by MolProbity [32]. Statistics of the model building are summarized in Table 1. All figures were prepared using Chimera [33] and ChimeraX. We used the PDBsum tool [34] to calculate protein–protein and protein–ligand interactions.

### 2.5. Protein Degradation Assays

A total of 4 μM TFAM was incubated with 0.8 μM human LonP1-His (hexamer concentration) in the reaction buffer containing 25 mM Tris-HCl (pH 7.5), 150 mM NaCl, 10 mM MgCl_2_, 1 mM DTT, and 5 mM ATP at 37 °C. At different time points, reaction aliquots were stopped by adding 5× SDS-polyacrylamide gel electrophoresis (SDS-PAGE) loading dye and heated at 95 °C for 5 min. Substrate degradation was assessed by SDS-PAGE and Coomassie Blue staining. Each experiment was performed in triplicate.

### 2.6. ATPase Activity Assays

The ATP hydrolysis activity of wild-type human LonP1 and the mutant (T564A-Y565A-V566A) was measured using a Malachite Green Phosphate Detection Kit (Beyotime, Shanghai, China) by quantifying the release of inorganic phosphate (Pi). The enzymatic reactions were carried out in a reaction buffer containing 25 mM Tris-HCl (pH 7.5), 150 mM NaCl, 10 mM MgCl_2_, and 1 mM DTT. Briefly, 0.8 μM of LonP1 (WT or 3A mutant, hexamer concentration) was pre-incubated with 5 mM ATP at 37 °C for 30 min, then 10 μL aliquots of the reaction mixture were withdrawn and immediately diluted 20-fold into 190 μL of double-distilled water. Subsequently, 70 μL of freshly prepared Malachite Green working reagent was added to the diluted samples in a 96-well plate. After a 30 min incubation at room temperature, the absorbance was measured at 630 nm using a microplate reader (Molecular Devices). The concentration of released Pi was determined utilizing a phosphate standard curve generated under identical conditions. All assays were performed in triplicate.

## 3. Results

### 3.1. Overall Structure of the Human LonP1 Protease Bound with TFAM

**Figure 1:**
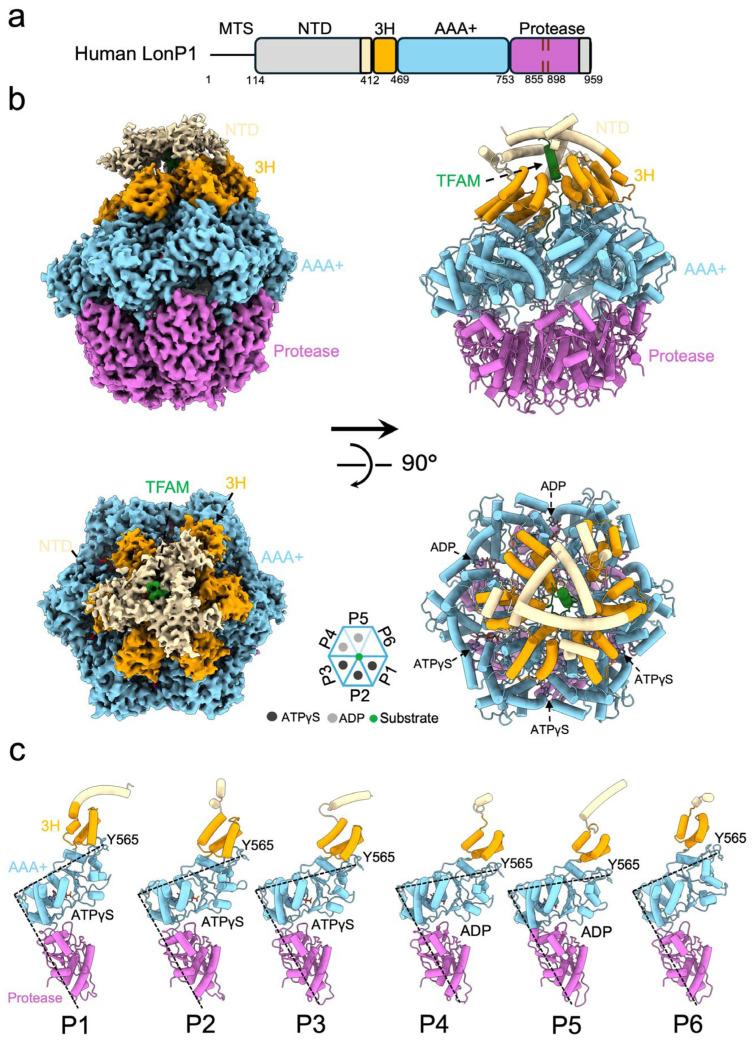
Architecture of the substrate-bound human LonP1 hexamer. (**a**) Domain organization of full-length human LonP1. The linear schematic highlights the mitochondrial targeting sequence (MTS), N-terminal domain (NTD, wheat), three-helix bundle (3H, gold), AAA+ ATPase module (cyan), and protease domain (purple). Key residue boundaries are indicated. (**b**) Orthogonal views of the cryo-EM density map (**left**) and atomic model (**right**) of the LonP1–TFAM complex. The structure is depicted in side view (**top**) and top view (**bottom**). Domains are colored and labeled corresponding to the scheme in (**a**). (**c**) The side view of six protomers is aligned by their protease domains and with distinct conformation states (P1–P6).

To determine the molecular architecture of the human LonP1-TFAM complex, we first assessed the quality and enzymatic competence of the recombinant full-length protein, which comprises an N-terminal domain (NTD), a central 3H domain, an AAA+ module, and a C-terminal protease domain (Figure 1a). Negative-stain transmission electron microscopy (TEM) of LonP1 purified from gel filtration confirmed a homogeneous particle distribution (Figure S1a,b,f), and in vitro degradation assays revealed robust proteolytic turnover of TFAM at 37 °C (Figure S1d,e,g), establishing that the purified LonP1 is fully active and primed for structural analysis.

A major challenge in visualizing protease-substrate complexes is the transient nature of the interaction. To overcome this, we strategically stalled the catalytic cycle by incubating LonP1 with TFAM in the presence of the slowly hydrolysable 5′-[γ-thio]-triphosphate (ATPγS) and the covalent inhibitor bortezomib. This approach successfully trapped the complex in a stable conformation, enabling structure determination by single-particle cryo-EM at 3.22 Å resolution (Table 1, Figure S2). The reconstruction unveiled a striking closed right-handed spiral organization of the hexamer (Figure 1b), characterized by an asymmetric arrangement of the six protomers and the ATPγS and ADP binding assignment of each promotor (Figure 1b,c) that corroborates a substrate-bound conformation.

Detailed inspection of the density map revealed domain-specific dynamic properties. While the AAA+ module and protease chamber were well-solved, allowing for the identification of typical elements such as the 3H domain and the classic Walker A and Walker B motifs (Figure S2c). Furthermore, chemical compounds added to the sample, including nucleotides ATPγS or ADP, were clearly discernible within the structure (Figure S2d). In contrast, the NTD displayed substantial conformational plasticity, evidenced by high local B-factors and smear density (Figure S2a,b). This observation suggests that the NTD likely undergoes dynamic motions to facilitate substrate capture, limiting modeling to a core helix of the triple-helix bundle. This unambiguous ligand definition and the asymmetric spiral architecture (Figure 1c) confirm that we have successfully trapped an active substrate-processing intermediate of human LonP1.

### 3.2. Molecular Basis of Substrate Recognition and Translocation in the Central Channel

**Figure 2:**
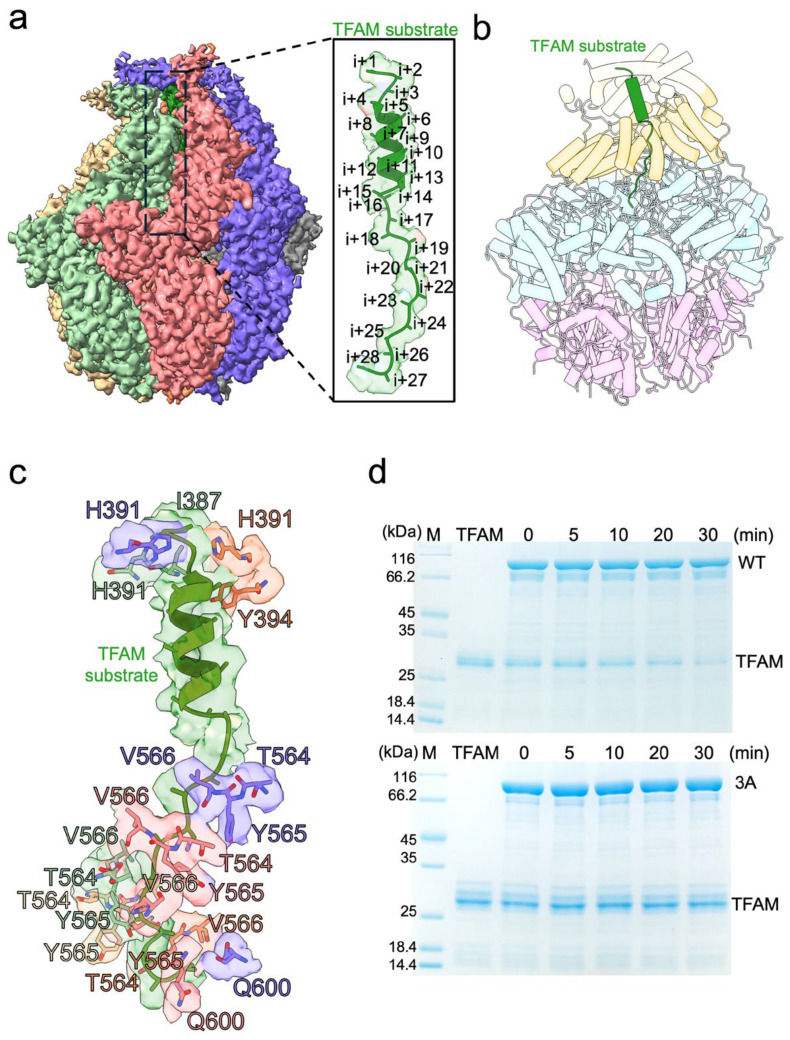
Structural analysis of substrate recognition and translocation. (**a**), Surface representation of the LonP1 hexameric channel engaged with the substrate. A zoom-in view (box) highlights the cryo-EM density of the translocating substrate polypeptide (green), spanning approximately 24 residues (labeled i+1 to i+28), threading through the central channel. (**b**) Side view of the atomic model illustrating the substrate (green) positioned within the central pore formed by the AAA+ spiral. (**c**) Molecular details of the LonP1–substrate interaction. Key pore-loop residues, including the conserved aromatic pairs and charged/polar from adjacent protomers, are shown arranged in a spiral staircase. (**d**) Proteolytic activity of wild-type (WT) and pore-loop mutant LonP1 variant (3A) against TFAM.

The high-resolution structure of the LonP1–TFAM complex enabled us to trace the path of substrate through the hexameric assembly. The translocated TFAM polypeptide spans approximately 28 residues, occupying the central channel formed by the spiral arrangement of the AAA+ ring (Figure 2a,b), but the remaining was invisible due to the dynamic and disordered structure. The substrate is primarily maintained in an extended helix-like conformation as it threads through the central axial pore to the 3H domain. To elucidate the mechanism of translocation, we analyzed the enzyme-substrate interface in detail. This interaction is primarily mediated by a conserved pore-loop triad comprising residues T564, Y565 and V566. These residues are arranged in a specific helical “staircase” configuration, creating a continuous grip on the substrate. The asymmetric, hand-over-hand arrangement of these aromatic and hydrophobic residues suggests they function as paddles to progressively pull the substrate into the degradation chamber. To validate the functional criticality of these structural elements, we performed site-directed mutagenesis on the pore-loop triad. We generated a triple mutant (T564A-Y565A-V566A, denoted as 3A) and performed its size exclusion chromatography and ATPase activity, and the results show that the mutant elutes in the same volume and comparable ATP hydrolysis activity as the wild type (Figure S1a–c). Then we assessed its activity via in vitro degradation assays. While the wild-type (WT) LonP1 exhibited robust degradation of TFAM over a 30 min time course, the 3A mutant showed a significant reduction in proteolytic turnover, rendering the enzyme nearly inactive. Collectively, these structural and biochemical data provide a mechanistic basis for LonP1 function, confirming that the helical coordination of pore-loop residues is essential for the recognition and translocation of mitochondrial substrates.

### 3.3. Structural Divergence of the N-Terminal Domain Organization

**Figure 3:**
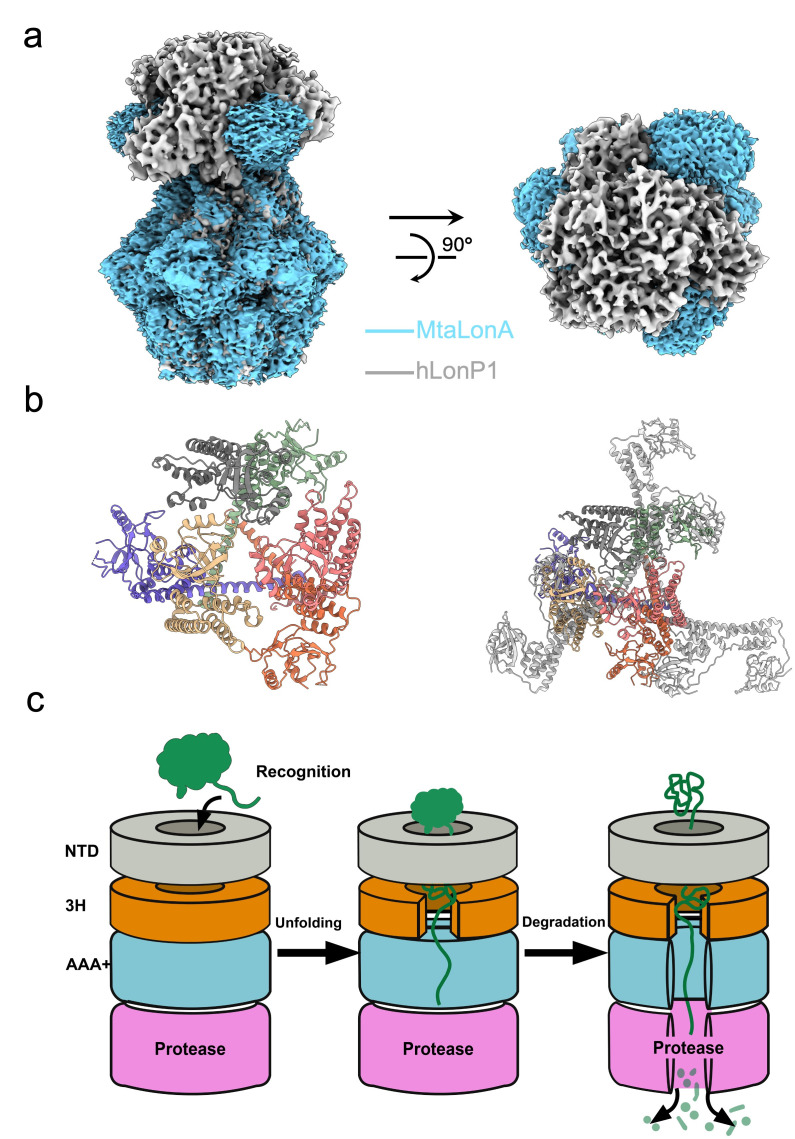
CompN terminal architecture of human LonP1 and proposed translocation mechanism. (**a**) comparison of cryo-EM map of human LonP1-TFAM complex and MtaLonA-substrate complex(EMD-35134), side view(left) and top view(right). (**b**) The N terminal domain of human LonP1 (left, generated from PDB:7NFY) and structural alignment between NTD of human LonP1 NTD and NTD of MtaLonA. (**c**) Schematic working model of human LonP1.

To elucidate the role of the N-terminal region in substrate regulation, we analyzed the quaternary organization of the human LonP1 NTD within the hexameric assembly. Although we did not build the atomic model of NTD due to the limitation of local resolution, superposition with previously published human LonP1 structure (PDB:7NFY) reveals a good coordination between their N-terminal domains and our experimental local map. Structural superposition of our human LonP1 complex with the bacterial homolog *Meiothermus taiwanensis* LonA (MtaLonA, PDB:7FD4, EMDB:31534) reveals a striking architectural divergence. While the ATPase and protease domains align closely, the N-terminal domains exhibit fundamentally different organizations (Figure 3a). The bacterial MtaLonA typically adopts an extended, splayed-out conformation, where the NTDs project radially away from the central axis, creating a wide-open vestibule. In sharp contrast, the human LonP1 NTD displays a significantly more compact and condensed architecture. The NTDs fold inward, tightly capping the ATPase ring to form a continuous, cage-like superstructure over the substrate entry pore (Figure 3b and Figure S3e). Despite this sterically compact arrangement, the local density for the human NTD is notably fragmented and diffused compared to the well-resolved AAA+ core, indicating inherent conformational plasticity. This suggests that while the human NTD forms a restrictive gate to prevent non-specific access, unlike the open bacterial system, it retains the dynamic flexibility necessary to recruit and manipulate specific mitochondrial substrates like TFAM.

### 3.4. Mechanism of Substrate Processing and Pathogenic Deregulation

Based on the staircase architecture and the distinct ligand states observed in our cryo-EM reconstruction, we propose a comprehensive working model for the human LonP1 catalytic cycle (Figure 3c). The process initiates with the substrate capture phase, where the intrinsically flexible NTDs act as dynamic tentacles to guide the substrates into the central channel. Following capture, the NTD mediates an initial unfolding of the substrate’s tertiary structure, ensuring that only a single protein chain threads through the restricted triangle entry pore toward the central channel. Subsequently, sequential ATP hydrolysis drives the AAA+ ring to execute a “hand-over-hand” pulling motion via the spiral pore-loops, mechanically thoroughly unfolding the polypeptide into a linear polypeptide and translocating it into the protease chamber for degradation. Thus, the LonP1 hexamer operates as a strictly coordinated bipartite machine, where the peripheral domains handle specificity and initial unfolding, while the central core powers processive translocation and degradation.

## 4. Discussion

Our cryo-EM reconstruction of the human LonP1-TFAM complex bridges a critical gap in understanding mitochondrial proteostasis by revealing that human LonP1 hexamer functions not merely as a passive proteolytic chamber, but as a dynamic search-and-shred paradigm. A defining feature of this structure is the architectural divergence of the N-terminal domain compared to bacterial homologs. While bacterial LonA typically adopts a splayed-out, open conformation to indiscriminately capture degron-tagged targets directly or with the assistant protein like LarA [35], we show that human LonP1 has evolved a compact, cage-like superstructure that tightly caps the ATPase ring. This structural consolidation likely functions as a molecular filter, restricting access to the catalytic chamber. This evolutionary adaptation is consistent with the stricter regulatory requirements of the human mitochondrial matrix, where LonP1 must discern specific regulatory factors (like TFAM) from a dense milieu of bystander proteins, preventing uncontrolled proteolysis.

Importantly, our structural and biochemical data support a model where LonP1 operates as a strictly coordinated bipartite machine. The peripheral NTD assembly does not merely serve as a passive docking site but acts as an active selective vestibule. Despite its compact quaternary organization, the local density of the NTD remains fragmented and flexible, suggesting an entropic mechanism. We propose that these flexible domains survey and capture folded substrates, mediating an initial unraveling of tertiary structures to thread a single polypeptide chain into the central pore, and this feature was also determined from another structure (PDB:9CC3). Once engaged, the substrate is handed off to the central AAA+ motor. Here, the conserved T564–Y565–V566 pore loop triad established by our mutagenesis data is essential for catalysis, which forms a spiral staircase that executes a powerful hand-over-hand pulling motion. This second stage ensures the thorough mechanical unfolding required for translocation into the protease chamber.

Protease covalent inhibitors are widely used in cryo-EM studies of AAA+ proteases [15,36]. A potential concern in our study is whether the use of the covalent inhibitor bortezomib to stall the proteolytic chamber might introduce structural artifacts, particularly regarding the spiral hexameric assembly. However, structural comparisons with previously reported human proteases alleviate this concern. When aligning our LonP1–TFAM complex with other substrate-bound AAA+ protease structures, including those captured with bortezomib and those stalled via catalytic mutations without inhibitors, the right-handed spiral staircase conformation of the AAA+ ring remains highly conserved (Figure S4). This strongly suggests that the spiral architecture is a bona fide physiological state driven primarily by ATP binding and substrate engagement within the central channel, rather than an allosteric artifact induced by inhibitor binding at the distant protease domain.

Indeed, the elucidation of this native recognition interface offers a new paradigm for therapeutic intervention. Previous efforts have largely relied on inhibiting the proteolytic active site, which risks off-target toxicity. The unique, compact architecture of the human NTD and its dynamic interface with the ATPase ring present novel allosteric pockets specific to the human enzyme. Targeting these regions could allow for the development of compounds that modulate substrate entry without abolishing basal housekeeping degradation. Furthermore, capturing this translocation-competent intermediate provides a structural template for designing Proteolysis Targeting Chimeras (PROTACs) that hijack the LonP1 machinery to degrade pathogenic proteins, expanding the druggable landscape of mitochondrial diseases.

It is important to note that our structure represents a post-engagement translocation state, where the substrate is already threaded. The initial collision complex between the globular NTD and a fully folded TFAM remains structurally elusive due to its inherent heterogeneity. Future studies utilizing time-resolved cryo-EM or specific cross-linking strategies will be crucial to visualize these fleeting pre-engagement conformations, further completing the kinematic movie of LonP1 function.

## Figures and Tables

**Table 1:** Cryo-EM collection, processing and model validation of human LonP1-TFAM.

Data Collection and Processing	Human LonP1-TFAM
Microscope	Titan Krios G3i
Voltage (kV)	300
Camera	Gatan K3
Magnification	105,000×
Pixel size (Å)	0.82
Total exposure (e^−^/Å^2^)	45
Exposure time (s)	1.7
Number of frames per exposure	30
Energy filter slit width (eV)	20
Data collection software	EPU 2.7
Number of exposures per hole	4
Defocus range (μm)	−1.8 to −3.0
Number of micrographs collected	4891
Number of micrographs used	4830
Number of initial particles	292,800
Symmetry	C1
Number of final particles	99,919
Resolution (0.143 gold standard FSC, Å)	3.22
Atomic model refinement	
Software	phenix-1.21.2
Clashscores, all atoms	6.74
Poor rotamers (%)	0.04
Favored rotamers (%)	98.70
Ramachandran outliers (%)	0.00
Ramachandran favored (%)	97.71
MolProbity score	1.44
Bad bonds (%)	0.00
Bad angles (%)	0.06

## Data Availability

Cryo-EM map of HuLonP1 bound to TFAM, with its associated atomic models, has been deposited in the Electron Microscopy Data Bank (EMD-68281) and the Protein Data Bank (PDB:22IB).

## Supplementary Materials

The following supporting information can be downloaded at: https://www.mdpi.com/article/10.3390/life16030478/s1, Figure S1: Purification and biochemical characterization of recombinant human LonP1 and TFAM. Figure S2: Single-particle cryo-EM analysis of the human LonP1-TFAM complex. Figure S3: Details of domain organization and ligand/substrate binding in the LonP1 complex. Figure S4: Comparison between other reported human LonP1 structures.
