# Supplementary material for: Structural Analysis of Human LonP1 Protease Bound with the Native Substrate
Source: Life (Basel). 2026 Mar 16;16(3):478. doi: 10.3390/life16030478 (PMC13027955; doi:10.3390/life16030478)
Supplement: Supplementary file 1 [file life-16-00478-s001.zip › life-4138914-supplementary.pdf]

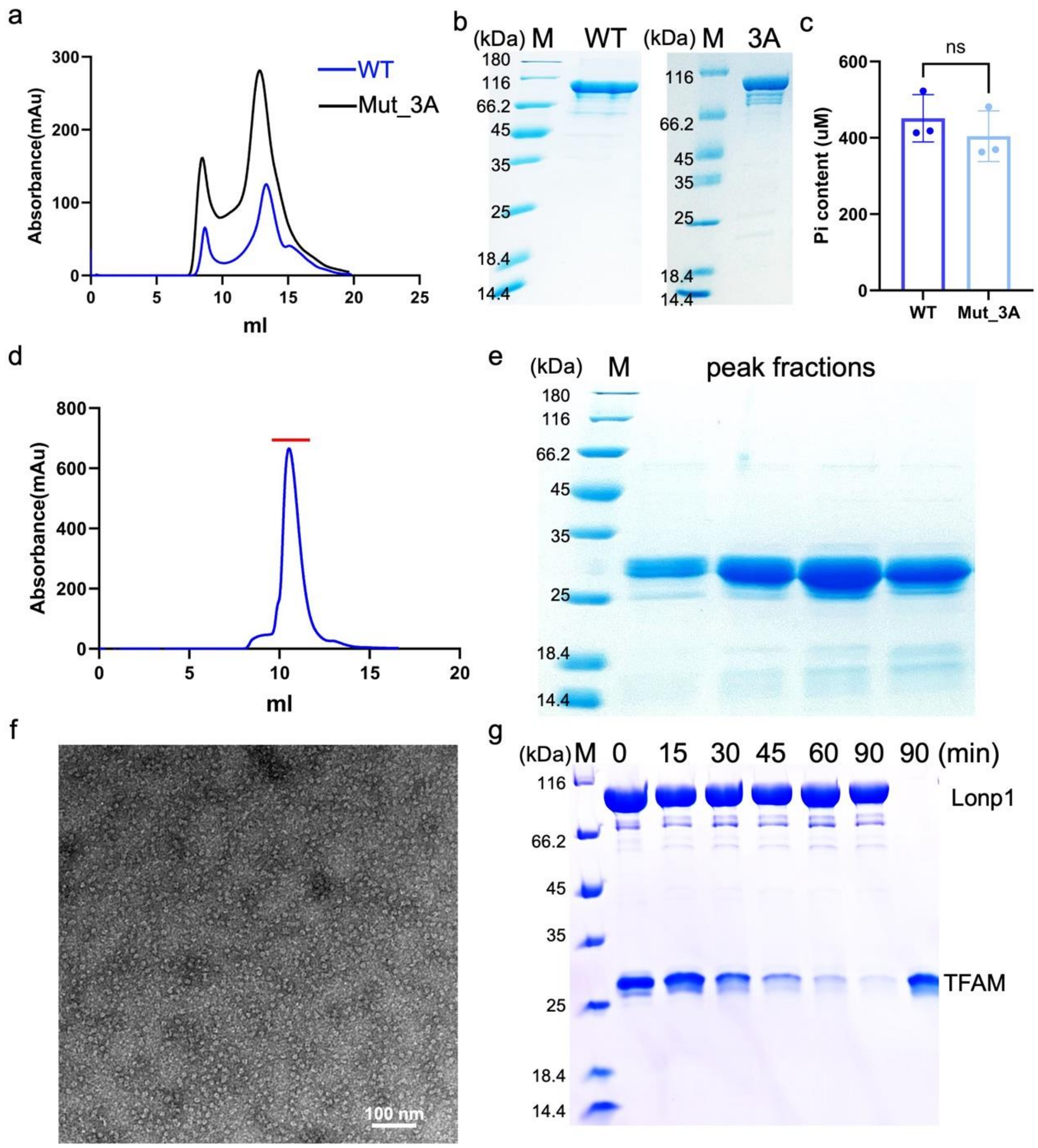

Figure S1. Purification and biochemical characterization of recombinant human LonP1 and TFAM.

a, Size -exclusion chromatography analysis of Human LonP1 protease.

b, SDS-PAGE analysis of the chromatography main peak fractions of Human LonP1 and pore-loop mutant(T564A-Y565A-V566A)..

c, Measurement of ATP hydrolysis activity of human LonP1 and pore-loop mutant(T564A-Y565A-V566A).

d, Size -exclusion chromatography analysis of TFAM.

e, SDS-PAGE analysis of the chromatography main peak fractions of TFAM.

f, Negatively stained transmission electron microscopy of the purified Human LonP1.

g, *In vitro* proteolytic activity assay of Human LonP1 to TFAM.

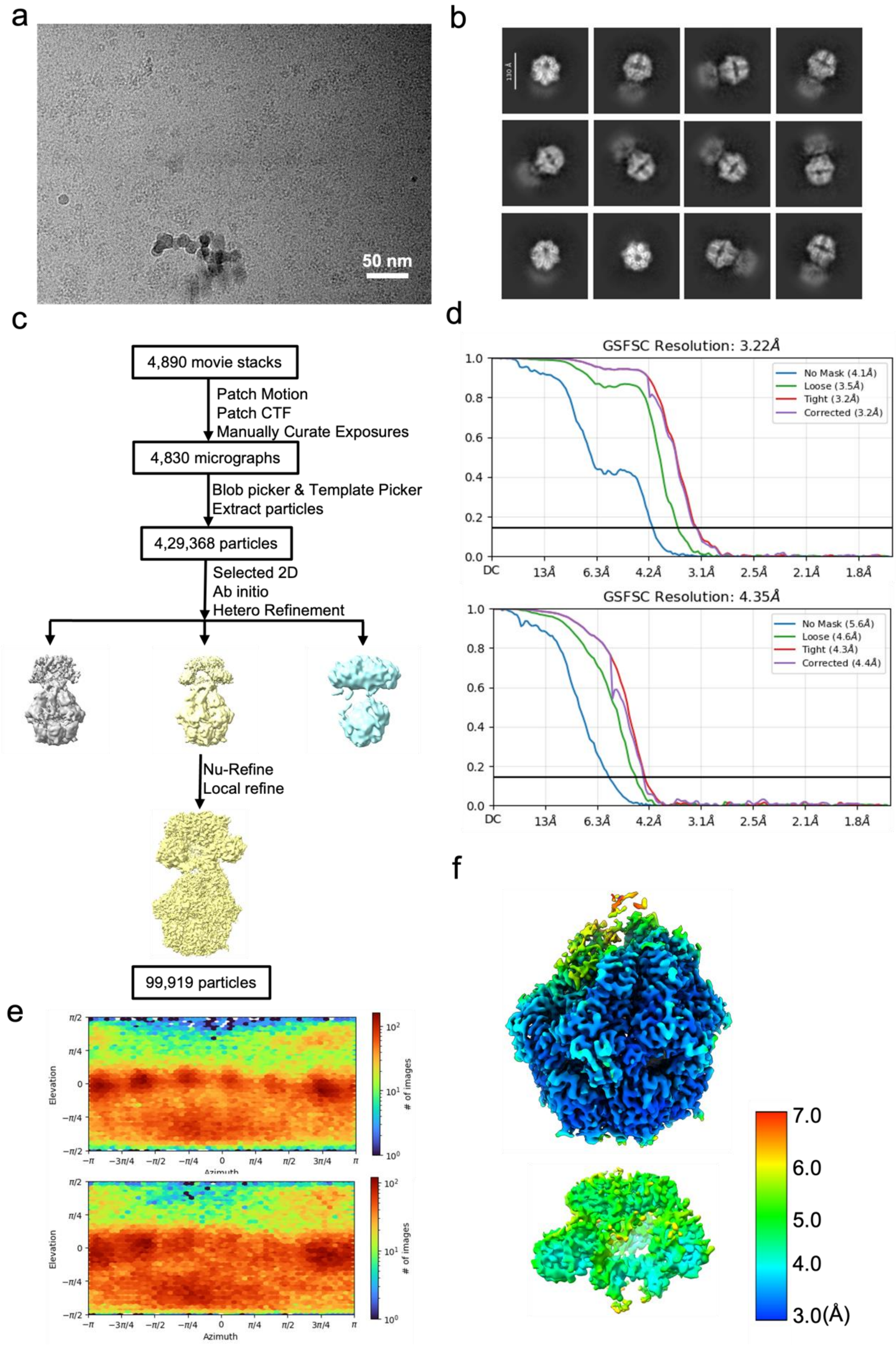

Figure S2. Single-particle cryo-EM analysis Human LonP1-TFAM complex.

a,Representative cryo-EM micrograph.

b,Reference-free 2D class averages.

c,Workflow of the cryo-EM data processing.

d,Gold standard FSC plots for the 3D reconstructions of the whole map(top: without N terminal domain, bottom: N terminal domain), calculated in cryoSPARC.
e, Euler angle distribution of the particle images (top: without N terminal domain, bottom: N terminal domain).
f ,The final map resolution is color coded for different regions(top: without N terminal domain, bottom: N terminal domain).

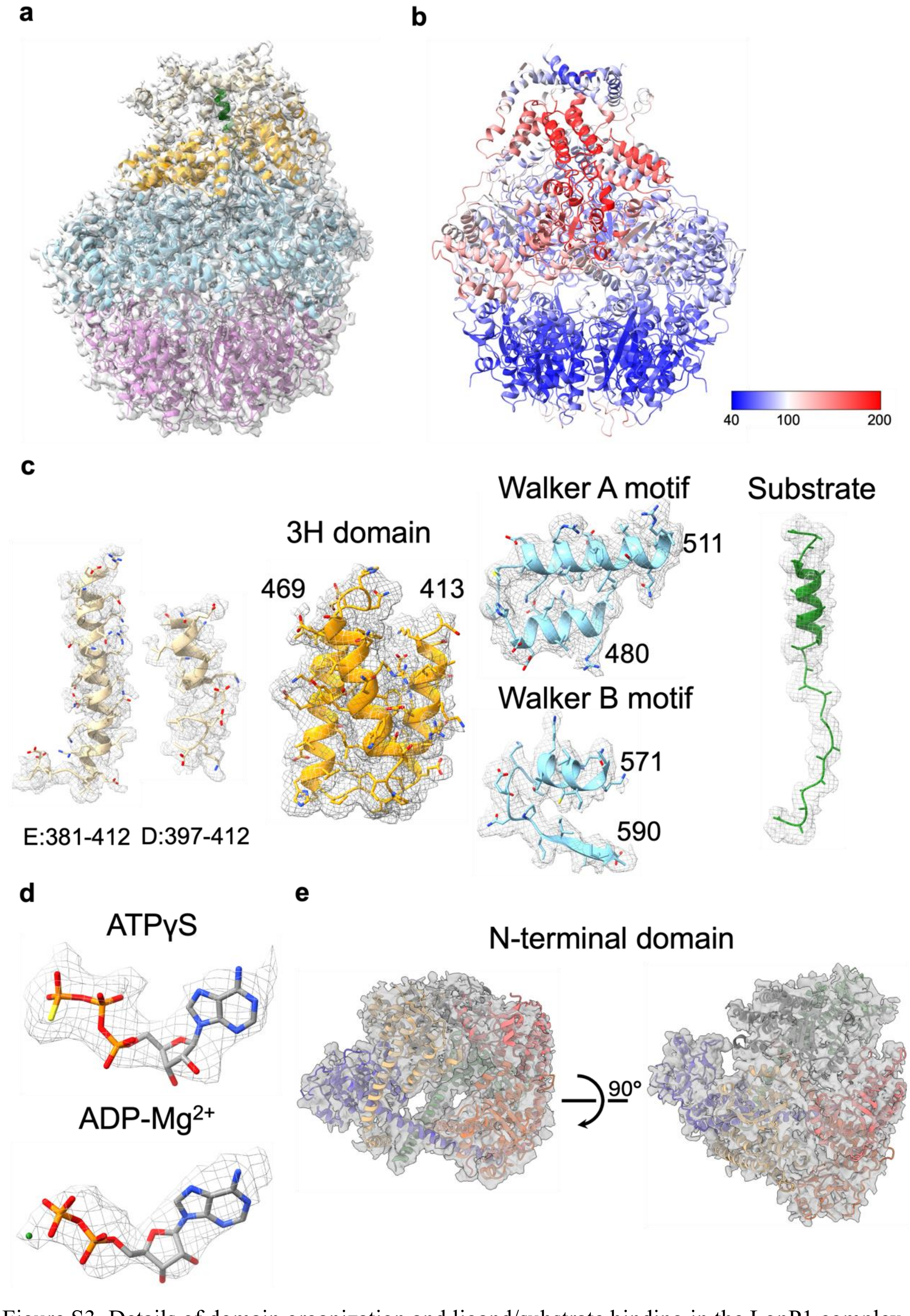

Figure S3. Details of domain organization and ligand/substrate binding in the LonP1 complex
a, Atomic model of Human LonP1-TFAM complex fitted into the cryo-EM map.
b, Local b factor estimation of modol of Human LonP1-TFAM complex.
c,CryoEM maps superimposed on atomic model of representative structures of Human LonP1-TFAM complex(N terminal domain colored in wheat,3H domain colored in gold,

Walker A motif and Walker B motif colored in cyan and substrate colored in forest green .
d, CryoEM maps superimposed on atomic model of representative ligands of Human LonP1-TFAM complex (magnesian ion colored in green).
e, The N terminal domain of the Human LonP1(PDB ID:7NFY) fitted into the calculated local cryo-EM map.

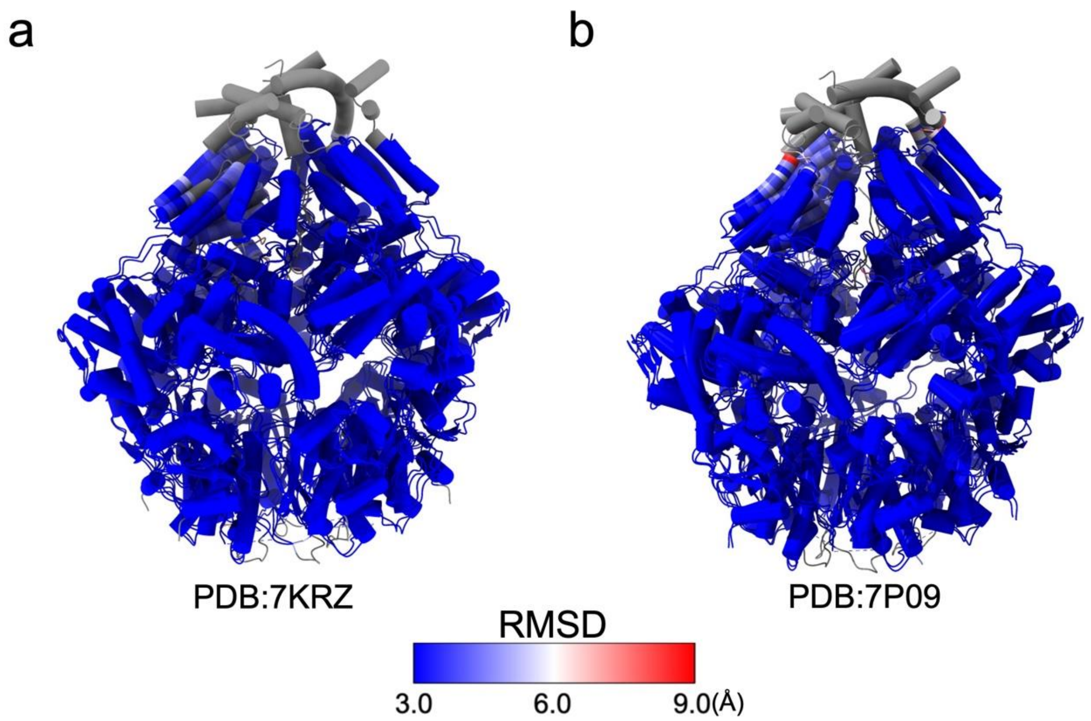

Figure S4. Comparison between other reported human LonP1 structures.

a, Comparison between human LonP1-Bortezomib with substrate (PDB:7KRZ), colored by C-alpha RMSD in ChimeraX.

b, Comparison between human LonP1with substrate (PDB:7P09), colored by C-alpha RMSD in ChimeraX.
